# Living Alone During COVID-19: Social Contact and Emotional Well-Being among Older Adults

**DOI:** 10.1093/geroni/igaa057.3479

**Published:** 2020-12-16

**Authors:** Yee To Ng, Karen Fingerman, Shiyang Zhang, Katherine Britt, Gianna Colera, Kira Birditt, Susan Charles

**Affiliations:** 1 The University of Texas at Austin, Austin, Texas, United States; 2 Texas State University, Austin, Texas, United States; 3 University of Michigan, Ann Arbor, Michigan, United States; 4 BSS, Irvine, California, United States

## Abstract

The COVID-19 outbreak and the associated physical distancing measures dramatically altered the social world for most older adults, but people who live alone may have been disproportionately affected. The current study examined how living alone was related to social contact and emotional well-being among older adults during the pandemic. Adults (N = 226) aged 69+ completed a brief survey assessing their living situation, social contact with different social partners (in person, by phone, electronically), and emotions during the morning, afternoon and evening the prior day. Older adults who live alone were less likely to see others in person or to receive or provide help, and reported less positive emotion the prior day than those who lived with others. Living alone was associated with more positive emotions concurrent with in-person contact. In contrast, phone contact was related to higher levels of negate affect among those living alone, but not among those who live with others. Findings suggest older adults who live alone may be more reactive to social contact during the COVID 19 outbreak than older adults who reside with others. In-person contact appears to confer distinct benefits not available via telephone contact, suggesting that possible interventions during the pandemic may work best with safe forms of in-person contact.

